# Exploring the Association between Diabetes Distress and Personality Traits: Insights from the Alternative DSM-5 Model for Personality Disorders

**DOI:** 10.31083/AP38760

**Published:** 2025-02-28

**Authors:** Judita Konečná, David Lacko, Eva Horová, Karel D. Riegel

**Affiliations:** ^1^3rd Department of Internal Medicine – Department of Endocrinology and Metabolism, 1st Faculty of Medicine, Charles University and General University Hospital, 128 08 Prague, Czech Republic; ^2^Department of Psychiatry, 1st Faculty of Medicine, Charles University and General University Hospital in Prague, 128 08 Prague, Czech Republic; ^3^2nd Department of Internal Medicine, St. Anne’s University Hospital, 602 00 Brno, Czech Republic; ^4^Institute of Psychology, Czech Academy of Sciences, 117 20 Brno, Czech Republic; ^5^Department of Addictology, 1st Faculty of Medicine, Charles University and General University Hospital in Prague, 128 08 Prague, Czech Republic

**Keywords:** diabetes mellitus, diabetes distress, diabetes distress scale, DDS, AMPD, personality traits

## Abstract

**Objective:**

Little is known about the association between subjectively experienced levels of diabetes distress (DD) and personality traits (PTs), even when levels of DD appear stable over time. This study aimed to use the Alternative Diagnostic and Statistical Manual of Mental Disorders, Fifth Edition (DSM-5) Model for Personality Disorders (AMPD) to associate specific maladaptive PTs with experienced DD and to describe differences in the constellation of PTs between people with type 1 diabetes (PWT1D) and type 2 diabetes (PWT2D).

**Methods:**

A total of 358 participants with diabetes mellitus (DM) (56.2% female, mean age 42.33 years, standard deviation (SD) = 14.33) were evaluated using the Diabetes Distress Scale (DDS) and the shortened 160-item version of the Personality Inventory for DSM-5 (PID-5). Psychometric properties of the DDS were evaluated first, then the association between DDS and PID-5 scores, and the differences between groups based on diabetes type and DD level, were analyzed.

**Results:**

Strong associations were found between the PID-5 Negative Affectivity (NEF) domain and the emotional burden (β = 0.852, *p*_Holm_ < 0.001) and regimen distress (β = 0.435, *p*_Holm_ = 0.006) DDS subscale scores. PWT1D had a higher level of personality pathology than PWT2D, as did participants with elevated levels of DD across most domains and facets of PID-5.

**Conclusions:**

Our findings suggest that attention should be paid to the level of NEF among people with diabetes in relation to their emotional burden and perception of regimen distress. We recommend a distinction between people based on their diabetes type. Implications for clinical practice and interventions for DD perceived through the lens of the dimensional DSM-5 PT model are discussed.

## Main Points

1. Personality traits, such as emotional lability, anxiousness, and separation insecurity, revealed among PWD by the Negative Affectivity domain are associated with high levels of DD aspects (emotional burden and regimen distress).

2. People with T1D scored higher on levels of personality pathology according to the PID-5 across broad domains and facets.

3. People with elevated levels of DD scored higher on the PID-5 across domains and facets in groups with both T1D and T2D.

4. Using the assessment of personality traits via PID-5, DD interventions could become more directly addressed and personalized, both in individual and group intervention planning.

## 1. Introduction

Psychological care is part of a new standard in a multidisciplinary approach to treatment of chronic diseases such as diabetes mellitus (DM). The psychological burden that people with diabetes (PWD) experience can increase the cost of treating the disease. Diabetes distress (DD)—negative stress specific to PWD caused by the demands of treatment, regimen, and the emotional burden often associated with DM [1]—is one of the psychological factors within PWD that may negatively influence disease management. Its high level, without proper intervention, can lead to diabetes burnout [2]. Although the level of DD appears stable over time [3], no specific aspect can account for this, which has led to the concept of personality traits (PTs). These are stable elements of personality that also play a role in diabetes management. PTs are connected to diabetes in two ways: some are protective and others are risk factors relating to adherence or treatment outcomes [4].

The Alternative Model for Personality Disorders (AMPD) was introduced into the Diagnostic and Statistical Manual of Mental Disorders, Fifth Edition (DSM-5) [5] as an option to approach personality disorders (PDs) from a dimensional perspective, as an alternative to the traditional categorical classification. The model assesses PDs based on impairments in personality functioning (criterion A) and maladaptive personality traits (criterion B). The level of personality functioning is assessed in four domains: self-directedness, identity, empathy, and intimacy. The level of pathological traits represents a constellation of maladaptive PTs based on five broad domains (Negative Affectivity [NEF], Detachment, Antagonism, Disinhibition, and Psychoticism) and 25 facets (e.g., anhedonia, anxiousness, callousness). PTs in this model are seen as maladaptive variants of the well-known Five-Factor Model (FFM) and are measured using the Personality Inventory for DSM-5 (PID-5) [6, 7].

Studies on PTs and diabetes have varied both in focus and results. For example, the FFM was used to find a connection between PTs and glycemic control, while high levels of neuroticism (i.e., NEF) were linked to decreased glycemic control [8]. Studies have also looked for a “typical” personality type of PWD (i.e., the constellation of specific PTs) and how to use it in clinical practice. For example, a MILES study found that possessing the type D personality (i.e., people with a tendency to experience negative emotions while not feeling free to express themselves) could be at a disadvantage in diabetes treatment [9]. Another study by Rouland *et al*. [10] revealed a connection between diabetes and the type A personality (i.e., people characterized as being ambitious, competitive, or impatient), which is more typical for patients with type 1 diabetes (T1D).

Nevertheless, being competitive and needing achievement has been seen as a protective factor in type 2 diabetes (T2D), with regard to diabetic foot ulcers [11]. Although PTs have been the object of many studies where researchers described the relationship between PTs and DM from various perspectives, the connection between perceived level of DD and PTs is not fully understood. It has been confirmed, however, that specific PTs such as neuroticism have an impact on the level of DD. Higher levels of neuroticism have been linked with the risk of experiencing sustained distress over time [12]. The level of DD also plays a mediating role in the association between neuroticism and other psychological aspects of diabetes - such as fear of hypoglycaemia [13].

Based on the above, it could be concluded that personality and its pathology require attention from healthcare professionals to identify interventions during DM treatment. The AMPD was recently used in a pilot study with people with type 2 diabetes (PWT2D) as a tool for therapeutic assessment. PWD, who received feedback based on an AMPD assessment, considered it helpful in managing T2D and decreasing their HbA1c levels [14]. Moreover, the usefulness of AMPD-based assessment of personality psychopathology in people with obesity has also been demonstrated [15], which speaks to the applicability of the DSM-5 dimensional model of PTs beyond the standard psychiatric setting, specifically in patients with various endocrinological problems. Considering that DM (especially T2D) is often associated with obesity [16], it could be hypothesized that maladaptive PTs may affect the subjective experience of diabetes treatment demands, like the treatment of obesity. The level of DD also appears to be stable over time. Therefore, the present study aimed to explore the association between the subjectively perceived level of DD and PTs, and to discuss the potential use of AMPD in treatment of PWD. We hypothesized that based on AMPD criteria, we would detect PWD with elevated DD, whose specific care needs might significantly impact the effectiveness of DD interventions and treatment adherence.

## 2. Material and Methods

### 2.1 Participants and Procedure

The total sample (*N* = 358) comprised outpatients recruited in collaboration with the Department of Endocrinology and Metabolism at the General University Hospital in Prague and participants in educational camps for patients with DM. The participants were included based on these criteria: the presence of a diagnosed T1D, T2D, gestational diabetes, or another type of diabetes (maturity-onset diabetes of the young, MODY, disease of pancreas, etc.); aged ≥18 years; and they could speak Czech and read, write, and understand the questionnaire text in Czech. Participation in the study was voluntary and anonymous for all respondents. Participants completed a self-report test battery comprised of a demographic questionnaire, Diabetes Distress Scale, and a shortened PID-5. The data were collected in a written or online format. Trained administrators (i.e., psychologists, educators, and tutors) conducted the data collection. The respondents were advised to get fully acquainted with all instructions before completing the test, and an administrator was also present to provide help if needed. The exact instructions were given to the participants who completed the online questionnaires. Access to the online form was shared during the educational camps and with a group of patients with DM on social media platforms.

### 2.2 Instruments

#### 2.2.1 Demographic Questionnaire

Participants completed a demographic questionnaire assessing their sex, age, educational level, marital status, diabetes type, current diabetes treatment, health complications, use of psychopharmaceuticals, and previous experience with psychiatric treatment.

#### 2.2.2 Diabetes Distress Scale 

Levels of DD were measured using the Diabetes Distress Scale (DDS). The DDS is a 17-item questionnaire using a Likert scale with items scored from 1 (no distress) to 6 (severe distress) capturing distress experienced during the last month [1]. The total DDS score and the scores on its dimensions (i.e., emotional burden, physician-related distress, regimen-related distress, and interpersonal-related distress) were evaluated using a mean score of <2.0, indicating no distress; a mean score from 2.0 to 2.9 indicating moderate distress; and a mean score ≥3.0, indicating high distress. Mean scores were used to distinguish high, moderate, or low distress for each item, four dimensions, and the total scale [17].

#### 2.2.3 Personality Inventory for DSM-5

PTs were measured using the PID-5. The scores are used to assess five broad domains and 25 facets [18]. The main PT domains are: NEF, Detachment, Antagonism, Disinhibition, and Psychoticism. Each domain comprises three facets (i.e., emotional lability, anxiousness, and separation insecurity for the NEF domain). Items are evaluated on a 4-point Likert scale from 0 (false or often false), to 3 (accurate or usually true). For this study, the shortened 160-item version of the PID-5 was used. According to Riegel *et al*. [19], this version shows better facet unidimensionality and is less demanding than the original 220-item version.

### 2.3 Statistical Analyses

This study’s data were analyzed in a structural equation modeling (SEM) framework, with a set of confirmatory factor analyses (CFAs) for evaluation of the DDS psychometric properties and with multiple regressions within SEM for assessing associations between the DDS and PID-5. All analyses were conducted in *R* [20]. To verify the factor structure of the DDS, we used a CFA to test the hierarchical model with four first-order and one second-order factor, which represented a general factor of the DDS. Next, the reliability of subscales was verified with Cronbach’s alpha and McDonald’s omega. The results of those analyses are described below. The regression analysis results were bootstrapped with 10,000 nonparametric bootstraps to obtain confidence intervals, estimates, and *p*-values of natural effects. Additionally, in this analysis, the PID-5 dimensions were inserted into the analysis as observed variables (as arithmetic means) to make the model parsimonious and less complex using item parceling (subset-item-parcel approach) [21]. This approach was necessary because the full PID-5 model, which includes five latent second-order factors, 25 latent first-order factors, and 160 indicators, was not feasible with our sample size. Two separate analyses (one with five broad domains of PID-5 as predictors and the second with all 25 facets of the PID-5 as predictors) were performed. Due to the vast amount of regression analyses, we adjusted the *p*-values with the Holm-Bonferroni method to reduce the risk of type I errors caused by multiple comparisons. To compare differences in PID-5 domains and facets between groups based on diabetes type (T1 vs T2) and DD level (overall score in DDS >2 vs DDS <2), Mann-Whitney tests with Holm-Bonferroni correction were used.

## 3. Results

### 3.1 Sample Characteristics

Of the (*N* = 358) respondents, 219 (61.2%) were diagnosed with T1D, 129 (36%) with T2D, seven (2%) with another diabetes type (MODY, a pancreas disorder), and two (0.5%) were diagnosed with gestational diabetes; 56.2% (*n* = 201) of the participants were female, the mean (M) age of the sample was 42.33 (SD [standard deviation] = 14.33, Me [median] = 42, IQR [interquartile range] = 26.75) years. The median body mass index (BMI) was 28.1 for men and 26.89 for women. The mean duration of DM was 12.9 (SD = 10.4, Me = 10, IQR = 14) years; 36.3% (*n* = 130) of the sample had experienced at least one chronic diabetes complication, while 48 participants (13.4%) reported more than one complication. Per cases of T1D, most of the respondents had been using an insulin pump (*n *= 90; 25.1%). Overall, 11.2% (*n* = 40) were treated with intensive insulin therapy (besides those with insulin pump) and 85 participants (23.7%) were treated with oral antidiabetics. The rest of the sample reported treatment with a combination of oral antidiabetics and injections (i.e., insulin once a day or incretin mimetics); 17.3% (*n* = 62) of the patients reported a history of psychiatric treatment and 9.8% (*n* = 35) were currently taking psychopharmaceuticals. In 28.8% (*n* = 103) of cases, the respondents were single, 53.6% (*n* = 191) were married, and 17.3% (*n *= 62) were divorced or widowed. Most respondents had completed secondary education (*n *= 183; 51.1%) and 133 (37.2%) had completed higher education or university. The mean (M) level of DD in the sample was 2.18 (SD = 0.95, Me = 1.82).

### 3.2 DDS Psychometric Properties

#### 3.2.1 DDS Factor Structure

In the first step, CFA was used to verify the original four-factor structure of the DDS proposed by Polonsky *et al*. [2] in the Czech sample. The proposed model showed satisfactory fit indices except for the root mean square error of approximation (RMSEA) *X*^2^ (113) = 285.976, *p* < 0.001, RMSEA = 0.082 [90% confidence interval (CI): 0.70, 0.093], standardized root mean squared residual (SRMR) = 0.070, comparative fit index (CFI) = 0.938, Tucker-Lewis index (TLI) = 0.925. Therefore, we analyzed the modification indices and allowed two residual correlations (between DDS8 and DDS12 and between DDS6 and DDS12) based on them. This model yielded satisfactory fit indices, *X*^2^ (111) = 240.580, *p* < 0.001, RMSEA = 0.071 [90% CI: 0.059, 0.084], SRMR = 0.053, CFI = 0.953, TLI = 0.943. According to a scaled chi-squared difference test, such improvement was statistically significant (Δ*X*^2^ = 51.60129, Δdf = 2, *p* < 0.001). Furthermore, all factor loadings were higher than 0.50 and, as shown in Table 1, the latent variables were highly correlated. The average variance extracted (AVE) was also higher than 0.50 on all subscales (see Table 1).

**Table 1.** **Correlations between DDS factors**.

	Emotional Burden	Physician Distress	Regimen Distress	Interpersonal Distress
EB	-			
PD	0.548***	-		
RD	0.774***	0.581***	-	
ID	0.667***	0.524***	0.687***	-

*** *p* < 0.001. DDS, Diabetes Distress Scale; EB, Emotional Burden; PD, Physician Distress; RD, Regimen Distress; ID, Interpersonal Distress.

Besides the four-factor model, the hierarchical model with four first-order and one second-order factor (representing a general factor of DDS) was also tested. This model yielded almost identical results, *X*^2^ (113) = 240.359, *p* < 0.001, RMSEA = 0.070 [90% CI: 0.058, 0.083], SRMR = 0.053, CFI = 0.954, TLI = 0.945, and thus both nested models did not differ significantly, Δ*X*^2^ = 0.40836, Δdf = 2, *p* = 0.815. Thus, the additional restrictions did not worsen the model fit.

Finally, the measurement invariance within the multigroup CFA of the scale between sex, DM types, and two age groups was performed. Metric and scalar invariances were established (see Appendix Table 6).

#### 3.2.2 DDS Reliability

In the next step, the reliability of subscales was verified with Cronbach’s alpha and McDonald’s omega. Both these indicators suggested good internal consistency (see Table 2).

**Table 2.** **Internal consistency of DDS**.

Coefficient	Emotional Burden	Physician Distress	Regimen Distress	Interpersonal Distress
α	0.917	0.877	0.864	0.915
ω	0.919	0.883	0.805	0.918

α, Cronbach’s alpha; ω, McDonald’s omega.

### 3.3 Association between DDS and PID-5

To analyze the associations between DDS and PID-5, we used the same DDS measurement model (i.e., four latent factors with the two correlated residuals), while the PID-5 factors were inserted into the structural model as the manifest variables. We performed two separate SEM analyses.

We analyzed the associations between the five PID-5 domains and four DDS factors in the first model. In the second model, we used all 25 PID-5 facets as predictors and one general second-order factor of DDS as an outcome (due to many regression analyses). Both models showed satisfactory fit indices, *X*^2^ (176) = 347.415, *p* < 0.001, RMSEA = 0.062 [90% CI: 0.052, 0.072], SRMR = 0.046, CFI = 0.947, TLI = 0.933; and *X*^2^ (561) = 4256.204, *p* < 0.001, RMSEA = 0.046 [90% CI: 0.040, 0.052], SRMR = 0.040, CFI = 0.914, TLI = 0.917, respectively. Complete analyses with 25 facets and four subscales of the DDS are shown in the Appendix (see Appendix Table 7).

**Fig. 1.**
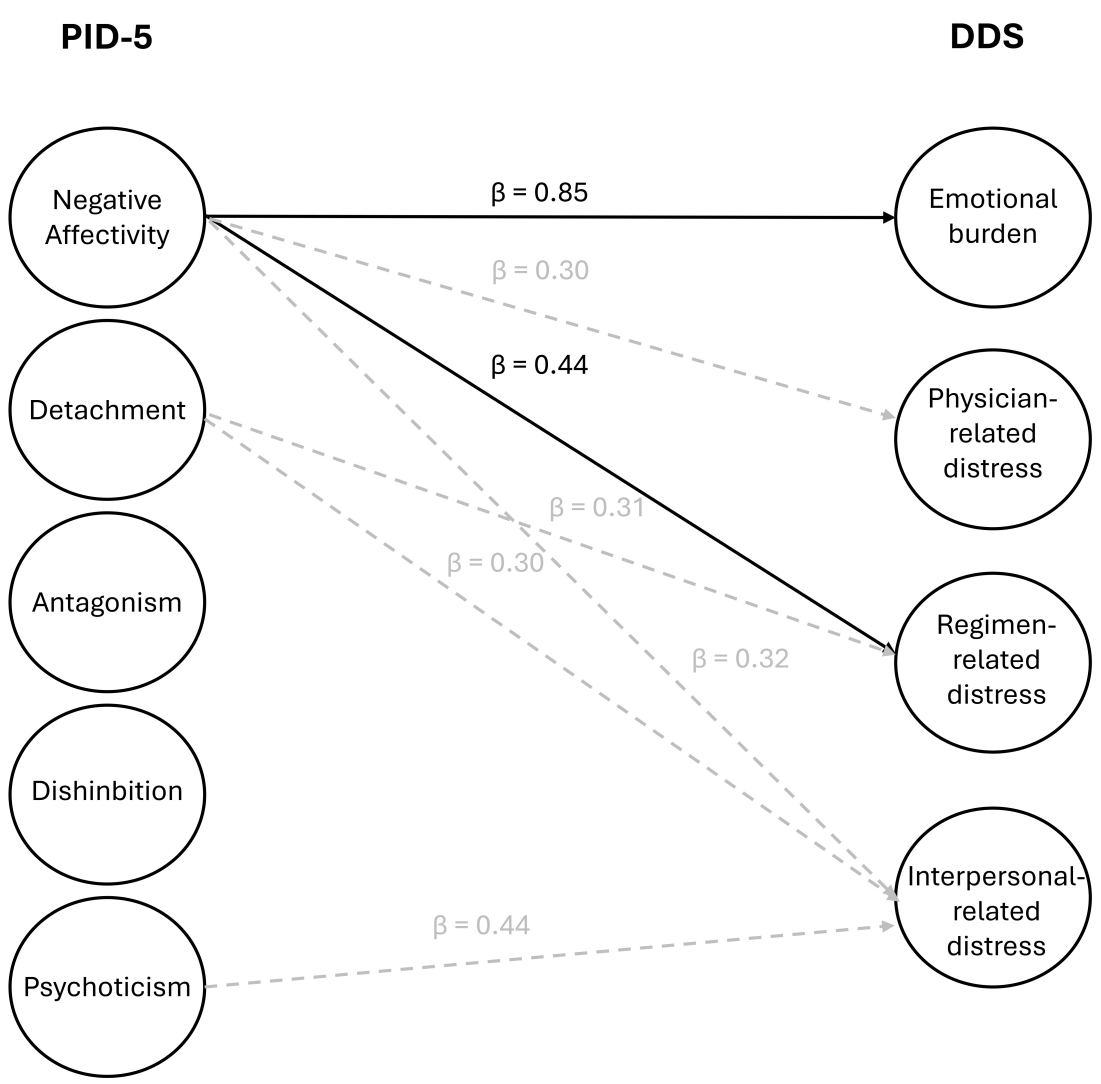
**Association between PID-5 domain and DDS subscale scores**.

Our findings (see Tables 3,4) suggest a strong association between the PID-5 domains of NEF and emotional burden (EB) (β = 0.852, *p* < 0.001) and a moderate association between the domains of NEF and regimen distress (RD) (β = 0.435, *p* = 0.006). Even though some other associations showed weak to moderate strength associations (see Fig. 1), they were statistically significant only without Holm-Bonferroni correction and were, therefore, not further investigated.

**Table 3.** **DDS factors predicted by broad domain of PID-5**.

Outcome	Predictor	β	95% CI	SE	*p*-value	*p*_Holm_
EB	Negative affectivity	0.852	0.634, 1.071	0.112	<0.001	<0.001
	Detachment	0.127	–0.108, 0.362	0.120	0.289	1
	Antagonism	–0.100	–0.394, 0.193	0.150	0.502	1
	Disinhibition	0.130	–0.188, 0.448	0.162	0.423	1
	Psychoticism	0.133	–0.178, 0.445	0.159	0.402	1
PD	Negative affectivity	0.299	0.047, 0.551	0.128	0.020	0.280
	Detachment	–0.024	–0.430, 0.382	0.207	0.908	1
	Antagonism	–0.020	–0.434, 0.393	0.211	0.923	1
	Disinhibition	0.316	–0.090, 0.722	0.207	0.127	1
	Psychoticism	0.119	–0.273, 0.511	0.200	0.551	1
RD	Negative affectivity	0.435	0.199, 0.671	0.112	<0.001	0.006
	Detachment	0.312	0.062, 0.562	0.128	0.015	0.225
	Antagonism	0.061	–0.255, 0.376	0.161	0.707	1
	Disinhibition	0.290	–0.021, 0.601	0.159	0.067	0.871
	Psychoticism	0.141	–0.196, 0.479	0.172	0.412	1
ID	Negative affectivity	0.321	0.083, 0.560	0.122	0.008	0.144
	Detachment	0.299	0.060, 0.538	0.122	0.014	0.224
	Antagonism	–0.044	–0.372, 0.283	0.167	0.790	1
	Disinhibition	–0.038	–0.398, 0.322	0.184	0.837	1
	Psychoticism	0.444	0.095, 0.793	0.178	0.013	0.221

β, standardized beta regression; CI, confidence intervals; SE, standard error; *p*, *p*-value; *p*_Holm_, *p*-value corrected by applying the Holm-Bonferroni method; PID-5, Personality Inventory for DSM-5.

**Table 4.** **DDS general factor predicted by 25 facets of PID-5**.

Outcome	Predictor	β	95% CI	SE	*p*-value	*p*_Holm_
DDS	Anxiousness	0.371	0.125, 0.617	0.126	0.003	0.075
	Emotional Lability	–0.060	–0.302, 0.182	0.124	0.627	1
	Separation Insecurity	0.038	–0.173, 0.248	0.107	0.726	1
	Anhedonia	0.142	–0.141, 0.426	0.145	0.325	1
	Intimacy Avoidance	–0.076	–0.243, 0.091	0.085	0.372	1
	Withdrawal	–0.072	–0.283, 0.140	0.108	0.505	1
	Deceitfulness	–0.013	–0.335, 0.308	0.164	0.936	1
	Grandiosity	0.148	–0.077, 0.372	0.114	0.197	1
	Manipulativeness	–0.035	–0.420, 0.351	0.197	0.860	1
	Distractibility	0.002	–0.233, 0.238	0.120	0.984	1
	Impulsivity	0.210	0.019, 0.401	0.098	0.031	0.713
	Irresponsibility	0.210	–0.145, 0.565	0.181	0.246	1
	Eccentricity	0.195	–0.058, 0.448	0.129	0.131	1
	Cognitive and Perceptual Dysregulation	–0.209	–0.608, 0.191	0.204	0.306	1
	Unusual Beliefs and Experiences	0.098	–0.121, 0.317	0.112	0.382	1
	Attention Seeking	–0.134	–0.325, 0.057	0.098	0.169	1
	Callousness	–0.192	–0.486, 0.101	0.150	0.199	1
	Depressivity	0.443	0.077, 0.809	0.187	0.018	0.432
	Hostility	0.148	–0.142, 0.439	0.148	0.317	1
	Perseveration	0.025	–0.252, 0.302	0.141	0.858	1
	Restricted Affectivity	0.019	–0.178, 0.216	0.101	0.847	1
	Rigid Perfectionism	–0.071	–0.253, 0.111	0.093	0.442	1
	Risk Taking	–0.032	–0.261, 0.198	0.117	0.788	1
	Submissiveness	–0.094	–0.250, 0.061	0.079	0.235	1
	Suspiciousness	0.049	–0.159, 0.256	0.106	0.645	1

β, standardized beta regression; CI, confidence intervals; SE, standard error; *p*, *p*-value; *p*_Holm_, *p*-value corrected by applying the Holm-Bonferroni method; DDS, Diabetes Distress Scale.

On the general DDS factor-level predicted by 25 PID-5 facets there was no association that was statistically significant after applying Holm-Bonferroni correction. The strongest association existed between anxiousness and the total level of DDS (β = 0.371, *p* = 0.075). There was also a moderate association between depressivity and DDS level (β = 0.443, *p* = 0.0432). The model with 25 facets of PID-5 and four factors of DDS yielded satisfactory fit indices, *X*^2^ (436) = 716.766, *p* < 0.001, RMSEA = 0.047 [90% CI: 0.041, 0.053], SRMR = 0.032, CFI = 0.927, TLI = 0.906. The only result that remained statistically significant after Holm-Bonferroni correction was between anxiousness and emotional burden (EB) (β = 0.559, *p* < 0.001; see Appendix Table 7).

### 3.4 Personality Traits, Diabetes Type, and Diabetes Distress

Additionally, we conducted Mann-Whitney tests to explore the differences in the distribution of PTs between T1D and T2D. The sample consisted of 219 people with type 1 diabetes (PWT1D) and 129 PWT2D. Statistically significant differences with small effect sizes were found between PWT1D and PWT2D in their NEF (*p* < 0.001, *r* = 0.28), Antagonism (*p* = 0.035, *r* = 0.14), Disinhibition (*p* = 0.001, *r* = 0.20), and Psychoticism (*p* = 0.001, *r* = 0.20) per the broad PID-5 domains (see Table 5). PWT1D scored higher in all domains. The differences in the level of facets are shown in the Appendix Table 8.

**Table 5.** **Differences between type 1 and type 2 diabetes and between levels of DD in personality traits according to PID-5 domains**.

Variable	Comparison	H-L	W	*p*_Holm_	r
Negative Affectivity	Type 1 vs. Type 2	0.359	18,896	<0.001	0.282
	DD <2 vs. DD >2	–0.627	6508	<0.001	0.503
Detachment	Type 1 vs. Type 2	0.010	14,352	0.803	0.013
	DD <2 vs. DD >2	–0.448	8332	<0.001	0.403
Antagonism	Type 1 vs. Type 2	0.083	16,497	0.017	0.141
	DD <2 vs. DD >2	–0.167	10,413	<0.001	0.290
Disinhibition	Type 1 vs. Type 2	0.194	17,452	0.001	0.197
	DD <2 vs. DD >2	–0.444	7536	<0.001	0.446
Psychoticism	Type 1 vs. Type 2	0.162	17,540	0.001	0.202
	DD <2 vs. DD >2	–0.378	7987	<0.001	0.422

H-L, Hodges-Lehmann estimate of the location parameter; W, W statistics; *p*_Holm_, *p*-value corrected by applying the Holm-Bonferroni method; *r*, rank-biserial correlation; DD, diabetes distress.

Following, again using the Mann-Whitney tests, we checked for differences in the groups based on DD level: participants with DD <2 (*n* = 219) versus participants with elevated DD >2 (*n* = 129). Differences were found among all the broad PID-5 domains with medium effect sizes (all *p*s < 0.001, *r*s varied between 0.29 and 0.50; see Table 5). In all cases, participants with DD >2 scored higher on all domains. Differences among all facets are shown in the Appendix Table 8.

## 4. Discussion

In line with the aim of the present study, we discovered a relationship between subjectively experienced levels of DD and maladaptive PTs according to the AMPD. In addition, we explored the differences in PTs according to the AMPD between groups of PWT1D and PWT2D and between groups with low and high levels of DD. We found a difference in levels of personality pathology between groups of T1D and T2D, with PWT1D scoring higher across domains and almost all facets of the PID-5. Higher levels of personality pathology were also reported by people with elevated DD.

### 4.1 DDS Psychometric Properties

The verification of the psychometric properties of the Czech version of the DDS was the first important step because, to the best of our knowledge, there has been no previous study using the Czech DDS reported. The Czech DDS version showed satisfactory psychometric properties in its factor structure, internal consistency, and measurement in variance between sex, diabetes types, and age. It is, therefore, approved for further clinical and research purposes in the Czech population with DM. Cronbach’s alpha of a 17-item scale was the same as that obtained by authors of the original version (0.93 [1]) and the Polish version [22].

### 4.2 Association between DDS and PID-5

One of the study’s main goals was to identify connections between subjectively-perceived DD and maladaptive PTs using the PID-5. Our findings suggest that the NEF broad domain of the PID-5 was significantly associated with two subscales of the DDS – EB and RD. To our knowledge, there has been no previous research on the DDS using the PID-5. Nevertheless, the AMPD trait model was aligned with the FFM, which was included in several studies [8, 9, 12]. The PID-5’s broad domain of NEF comprises three facets (anxiousness, emotional lability, separation insecurity) which might be linked to the FFM’s neuroticism domain. From this perspective, our results confirmed the findings from a MILES study [9] and a study from Sanatkar *et al*. [12]. In the former, the tendency to experience negative emotions was negatively connected with treatment demands (i.e., burden of diabetes). Conversely, the second study referred to the level of neuroticism and its relation to the level of DD. In Sanatkar *et al*.’s [12] study, the level of neuroticism affected more than one component of DD (including physician distress, EB, and RD). In our research, NEF affected all DD components. However only EB and RD were affected significantly.

### 4.3 Personality Traits, Diabetes Type, and Diabetes Distress

Our findings were also supported by differences found between groups based on their overall levels of DD, as participants with elevated DD scored higher on pathological PTs. Moreover, previous findings regarding the suitability of the PID-5 for personality assessment in patients with primarily endocrinological illnesses support these findings to some extent, as people with chronic diseases may experience feelings of loss and guilt related to a sense of self-negativity associated with a body that is symbolically damaged by chronic illness [23]. Some studies have supported the association between self-blame and poorer quality of life in PWD, as well as between increased levels of guilt and a tendency to cope through behavioral withdrawal, with negative implications for health outcomes [24].

In the present study, PWT1D reached statistically significantly higher levels of personality pathology among all broad domains of the PID except detachment, and most facets (17 out of 25) except anhedonia, withdrawal, grandiosity, manipulativeness, unusual beliefs and experiences, callousness, restricted affectivity, and risk-taking. T1D patients who score higher compared with T2D are usually those diagnosed with diabetes in childhood; the disease becomes part of their self-concept and affects their self-esteem, which is associated with emotional stability, EB of diabetes, or depressive symptoms [25]. Therefore, a negative self-concept may be associated with DD levels in adulthood. We also discovered that participants with higher DD showed statistically significantly higher levels on all broad domains of the PID-5 and across almost all facets (23 out of 25), except for risk-taking and attention-seeking.

Conversely, higher levels of risk-taking and intimacy avoidance in T2D were consistent with the findings of Riegel *et al*. [15], where these facets were elevated at the clinical level in a sample of pre-bariatric patients. As T2D is a common comorbidity of obesity, we might have expected some similarities in levels of personality pathology in our sample. Scores of maladaptive PTs were lower in our sample, but this may have been influenced by the fact that the average BMI in our sample was in the overweight range.

### 4.4 Implications for Practice

In considering the results from a systematic review and meta-analysis of effective interventions for reducing DD [26] where the effect sizes were low, we might answer the question of why a well-educated patient is still non-compliant. Sometimes, more practical tools are insufficient to achieve the desired result. In addition, according to Samadi *et al*. [27], behavioral interventions or education can increase patients’ self-concept and self-esteem. However, there are still cases in which systematic psychotherapy should be considered. It was proven by using the AMPD in the study of Huprich *et al*. [14] that receiving therapeutic assessment and guidance to understand how the level of personality functioning and pathological PTs might influence the diabetes management of PWT2D helped to decrease the level of HbA1c.

Understanding the emotion regulation skills associated with NEF according to the PID-5 might be helpful in DD reduction programming, as confirmed by Coccaro *et al*. [28]. Moreover, it could explain DD’s stability over time as described earlier [3]. Our results also showed that DD is associated with PTs and those with elevated DD scored higher overall on PTs. DD and other negative feelings such as shame, anger, self-blame, or fear have also been linked to a state of damaged ego, creating a transference-countertransference dynamic in which elements personal to both client and therapist interact, which may be helpful in recognizing relationships with healthcare providers when behavioral interventions are not working [16, 23]. Using the AMPD and giving feedback to the PWD can help them understand themselves and bring therapeutic effects, as shown by Huprich *et al*. [14].

### 4.5 Limitations

The present findings should be viewed in the context of certain limitations, which may inspire further research. From the perspective of the overall level of personality psychopathology within DM patients, the battery of tests should be enriched with specific instruments measuring the AMPD’s criterion A (i.e., the level of personality functioning), as well. However, each added inventory makes the battery of tests more demanding and time-consuming. This could partially be solved by using a shorter PID-5 form, which has proved sufficient in capturing AMPD criterion B (i.e., PTs) per the level of broad domains [29], and by adding a screening tool focused on the level of personality functioning (LPFS), like the LPFS-Brief Form [29]. It also seems essential to consider the subjectivity of the participants’ statements, as both tools used were self-report measures. However, the presented study was exploratory and pioneering regarding DD in the Czech Republic. Future investigations might use larger samples and batteries of tests that focus on treatment adherence outcomes, patient compliance, and assessment for symptoms of depression and anxiety, as elevated scores on the DDS are usually associated with being depressed and having poorer self-care and a lower quality of life [2]. Nevertheless, in line with Ehrenthal *et al*. [30], our results support the finding that the dimensional models of personality pathology and its screenings can help detect specific groups of “difficult to treat” patients who might benefit from psychological support in the diabetes treatment process.

## 5. Conclusions

Overall, we found that some PTs were associated with the subjective experiential level of DD. Therefore, not only DD screening but also personality assessment could be helpful in the evaluation of the psychological aspects of experiencing diabetes. Particular attention should be paid to the level of NEF, which includes traits of emotional lability, anxiousness, and separation insecurity associated with EB and perception of RD. Moreover, this study found the DDS to be a reliable instrument for research and clinical practice with Czech DM patients. An assessment of personality before intervening for DD might help address and personalize the treatment. When planning the intervention, the diabetes type should be considered. Furthermore, our findings may reflect that some people with specific accentuated PTs may perceive a diagnosis of DM to be more of a burden, especially T1D patients. However, before incorporating our findings into practical interventions for PWD, further research must be performed.

## Availability of Data and Materials

The datasets analysed in the study are available from the corresponding author on reasonable request.

## Appendix

See Tables 6,7,8.

**Table 6.** **Measurement invariance of DDS**.

Group	Level	*RMSEA *[90%* CI*]	*SRMR*	*CFI*	*TLI*	ΔR*MSEA*	Δ*SRMR*	ΔC*FI*	ΔT*LI*
Gender	Configural	0.079 [0.066, 0.092]	0.060	0.942	0.928	-	-	-	-
	Metric	0.080 [0.067, 0.092]	0.079	0.937	0.927	0.001	0.019	0.005	0.001
	Scalar	0.079 [0.067, 0.091]	0.079	0.935	0.929	–0.001	0.000	0.002	–0.002
Age	Configural	0.089 [0.077, 0.102]	0.070	0.924	0.907	-	-	-	-
	Metric	0.086 [0.073, 0.099]	0.074	0.925	0.914	–0.003	0.004	–0.001	–0.007
	Scalar	0.084 [0.072, 0.096]	0.075	0.925	0.917	–0.002	0.001	0.000	–0.003
DM type	Configural	0.083 [0.071, 0.095]	0.065	0.933	0.918	-	-	-	-
	Metric	0.083 [0.071, 0.094]	0.078	0.931	0.920	0.000	0.013	0.002	0.002
	Scalar	0.082 [0.071, 0.094]	0.079	0.927	0.920	0.001	0.001	0.004	0.000

* note: = RMSEA, Root mean square error of approximation; CI, Confidence intervals; SRMR, standardized root mean square residual; CFI, Comparative fit index; TLI, Tucker–Lewis index; Δ, delta (change).

**Table 7.** **Regression analyses**.

Outcome	Predictor	β	95% CI	SE	*p*-value	*p*_Holm_
Emotional Burden	Anxiousness	0.559	0.340, 0.778	0.112	<0.001	<0.001
	Emotional Lability	–0.072	–0.315, 0.171	0.124	0.560	1
	Separation Insecurity	0.031	–0.179, 0.241	0.107	0.775	1
	Anhedonia	0.046	–0.205, 0.296	0.128	0.720	1
	Intimacy Avoidance	–0.117	–0.284, 0.050	0.085	0.170	1
	Withdrawal	–0.053	–0.258, 0.152	0.105	0.612	1
	Deceitfulness	–0.060	–0.388, 0.268	0.168	0.723	1
	Grandiosity	0.224	0.018, 0.431	0.105	0.033	1
	Manipulativeness	–0.177	–0.520, 0.173	0.177	0.325	1
	Distractibility	–0.024	–0.255, 0.208	0.118	0.842	1
	Impulsivity	0.248	0.063, 0.432	0.094	0.009	0.882
	Irresponsibility	0.066	–0.275, 0.407	0.174	0.705	1
	Eccentricity	0.115	–0.127, 0.356	0.123	0.352	1
	Cognitive and Perceptual Dysregulation	–0.167	–0.549, 0.214	0.195	0.390	1
	Unusual Beliefs and Experiences	0.034	–0.178, 0.246	0.108	0.751	1
	Attention Seeking	–0.034	–0.216, 0.147	0.093	0.710	1
	Callousness	–0.119	–0.407, 0.170	0.147	0.420	1
	Depressivity	0.410	0.080, 0.740	0.169	0.015	1
	Hostility	0.115	–0.158, 0.388	0.139	0.411	1
	Perseveration	0.002	–0.273, 0.276	0.140	0.991	1
	Restricted Affectivity	0.029	–0.165, 0.224	0.099	0.767	1
	Rigid Perfectionism	–0.034	–0.217, 0.149	0.093	0.716	1
	Risk Taking	–0.065	–0.281, 0.152	0.110	0.558	1
	Submissiveness	–0.113	–0.269, 0.043	0.079	0.155	1
	Suspiciousness	0.088	–0.124, 0.300	0.108	0.416	1
Physician Distress	Anxiousness	0.169	–0.084, 0.423	0.129	0.191	1
	Emotional Lability	–0.021	–0.292, 0.250	0.138	0.881	1
	Separation Insecurity	0.001	–0.251, 0.253	0.129	0.996	1
	Anhedonia	0.209	–0.135, 0.552	0.175	0.234	1
	Intimacy Avoidance	–0.168	–0.394, 0.057	0.115	0.143	1
	Withdrawal	–0.259	–0.483, –0.035	0.114	0.024	1
	Deceitfulness	–0.188	–0.567, 0.191	0.193	0.332	1
	Grandiosity	0.114	–0.189, 0.418	0.155	0.460	1
	Manipulativeness	0.259	–0.294, 0.812	0.282	0.359	1
	Distractibility	0.154	–0.099, 0.406	0.129	0.233	1
	Impulsivity	0.148	–0.074, 0.369	0.113	0.191	1
	Irresponsibility	0.131	–0.264, 0.525	0.201	0.516	1
	Eccentricity	0.225	–0.056, 0.506	0.144	0.117	1
	Cognitive and Perceptual Dysregulation	–0.216	–0.723, 0.290	0.258	0.403	1
	Unusual Beliefs and Experiences	0.026	–0.214, 0.267	0.123	0.832	1
	Attention Seeking	–0.280	–0.483, –0.078	0.103	0.007	0.693
	Callousness	0.065	–0.298, 0.428	0.185	0.726	1
	Depressivity	0.024	–0.426, 0.474	0.229	0.917	1
	Hostility	0.096	–0.247, 0.440	0.175	0.582	1
	Perseveration	0.033	–0.317, 0.382	0.178	0.854	1
	Restricted Affectivity	0.092	–0.162, 0.346	0.129	0.477	1
	Rigid Perfectionism	–0.081	–0.301, 0.139	0.112	0.469	1
	Risk Taking	–0.003	–0.278, 0.282	0.140	0.982	1
	Submissiveness	–0.144	–0.325, 0.037	0.092	0.119	1
	Suspiciousness	0.178	–0.081, 0.437	0.132	0.178	1
Regimen Distress	Anxiousness	0.146	–0.078, 0.371	0.114	0.201	1
	Emotional Lability	–0.083	–0.330, 0.165	0.126	0.512	1
	Separation Insecurity	0.035	–0.191, 0.260	0.115	0.762	1
	Anhedonia	0.179	–0.137, 0.495	0.161	0.266	1
	Intimacy Avoidance	0.001	–0.166, 0.168	0.085	0.992	1
	Withdrawal	–0.070	–0.291, 0.151	0.113	0.533	1
	Deceitfulness	0.085	–0.262, 0.433	0.177	0.630	1
	Grandiosity	–0.041	–0.275, 0.194	0.120	0.735	1
	Manipulativeness	0.135	–0.324, 0.594	0.234	0.564	1
	Distractibility	–0.054	–0.309, 0.200	0.130	0.676	1
	Impulsivity	0.159	–0.041, 0.360	0.102	0.120	1
	Irresponsibility	0.373	–0.008, 0.753	0.194	0.055	1
	Eccentricity	0.157	–0.117, 0.430	0.139	0.262	1
	Cognitive and Perceptual Dysregulation	–0.160	–0.569, 0.249	0.209	0.443	1
	Unusual Beliefs and Experiences	0.077	–0.157, 0.310	0.119	0.521	1
	Attention Seeking	–0.117	–0.334, 0.099	0.111	0.288	1
	Callousness	–0.254	–0.570, 0.062	0.161	0.115	1
	Depressivity	0.504	0.094, 0.914	0.209	0.016	1
	Hostility	0.186	–0.124, 0.496	0.158	0.239	1
	Perseveration	0.058	–0.243, 0.360	0.154	0.704	1
	Restricted Affectivity	0.077	–0.142, 0.296	0.112	0.492	1
	Rigid Perfectionism	0.105	–0.333, 0.080	0.105	0.229	1
	Risk Taking	0.002	–0.241, 0.245	0.124	0.985	1
	Submissiveness	–0.026	–0.195, 0.142	0.086	0.760	1
	Suspiciousness	–0.055	–0.282, 0.172	0.116	0.635	1
Interpersonal Distress	Anxiousness	0.026	–0.218, 0.269	0.124	0.837	1
	Emotional Lability	0.042	–0.228, 0.312	0.138	0.761	1
	Separation Insecurity	0.043	–0.191, 0.278	0.120	0.718	1
	Anhedonia	0.147	–0.181, 0.476	0.167	0.378	1
	Intimacy Avoidance	0.047	–0.158, 0.253	0.105	0.651	1
	Withdrawal	0.046	–0.220, 0.312	0.136	0.735	1
	Deceitfulness	0.065	–0.308, 0.438	0.190	0.732	1
	Grandiosity	0.134	–0.132, 0.401	0.136	0.323	1
	Manipulativeness	–0.106	–0.520, 0.307	0.211	0.614	1
	Distractibility	0.061	–0.231, 0.353	0.149	0.681	1
	Impulsivity	0.058	–0.147, 0.264	0.105	0.578	1
	Irresponsibility	0.181	–0.198, 0.559	0.193	0.350	1
	Eccentricity	0.270	–0.002, 0.541	0.138	0.051	1
	Cognitive and Perceptual Dysregulation	–0.199	–0.655, 0.258	0.233	0.393	1
	Unusual Beliefs and Experiences	0.231	–0.061, 0.524	0.149	0.122	1
	Attention Seeking	–0.177	–0.384, 0.030	0.106	0.094	1
	Callousness	–0.262	–0.598, 0.075	0.172	0.128	1
	Depressivity	0.315	–0.106, 0.735	0.214	0.142	1
	Hostility	0.055	–0.276, 0.384	0.169	0.743	1
	Perseveration	–0.002	–0.316, 0.312	0.160	0.990	1
	Restricted Affectivity	–0.119	–0.363, 0.124	0.124	0.336	1
	Rigid Perfectionism	–0.021	–0.251, 0.210	0.118	0.859	1
	Risk Taking	–0.004	–0.259, 0.251	0.130	0.976	1
	Submissiveness	–0.040	–0.219, 0.139	0.092	0.661	1
	Suspiciousness	–0.013	–0.263, 0.237	0.128	0.918	1

* note: β, standardized beta regression; CI, confidence intervals; SE, standard error; *p*, *p*-value; *p*_Holm_, *p*-value corrected by Holm-Bonferroni method.

**Table 8.** **Differences between type 1 and type 2 diabetes and between levels of DD in personality traits according to PID-5 facets**.

Variable	Comparison	H-L	W	*p*_Holm_	r
Anxiousness	Type 1 vs. Type 2	00.286	18,072	0.000	0.234
	DD <2 vs. DD >2	–00.714	66,910.0	0.000	0.494
Emotional Lability	Type 1 vs. Type 2	00.400	18,596.5	0.000	0.265
	DD <2 vs. DD >2	–00.800	76,180.5	0.000	0.443
Separation Insecurity	Type 1 vs. Type 2	00.333	17,860.5	0.001	0.222
	DD <2 vs. DD >2	–00.333	100,410.5	0.000	0.311
Anhedonia	Type 1 vs. Type 2	00.143	16,070,5	0.219	0.115
	DD <2 vs. DD >2	–00.571	76,060.5	0.000	0.444
Intimacy Avoidance	Type 1 vs. Type 2	00.200	10,886	0.006	0.193
	DD <2 vs. DD >2	–00.200	132,060.5	0.028	0.138
Withdrawal	Type 1 vs. Type 2	00.000	15,211	0.458	0.065
	DD <2 vs. DD >2	–00.400	94,350.5	0.000	0.344
Deceitfulness	Type 1 vs. Type 2	00.167	16,609.5	0.046	0.150
	DD <2 vs. DD >2	–00.167	108,340.5	0.000	0.271
Grandiosity	Type 1 vs. Type 2	00.000	15,682	0.360	0.096
	DD <2 vs. DD >2	–00.333	115,060.5	0.000	0.240
Manipulativeness	Type 1 vs. Type 2	00.000	15,356	0.402	0.080
	DD <2 vs. DD >2	–00.250	130,700.0	0.011	0.159
Distractibility	Type 1 vs. Type 2	00.250	17,330.5	0.007	0.190
	DD <2 vs. DD >2	–00.625	76,030.5	0.000	0.444
Impulsivity	Type 1 vs. Type 2	00.250	17,565.5	0.003	0.205
	DD <2 vs. DD >2	–00.500	95,420.5	0.000	0.340
Irresponsibility	Type 1 vs. Type 2	00.143	17,091	0.014	0.177
	DD <2 vs. DD >2	–00.286	93,840.0	0.000	0.348
Eccentricity	Type 1 vs. Type 2	00.182	18,190	0.000	0.242
	DD <2 vs. DD >2	–00.545	78,280.5	0.000	0.432
Cognitive and Perceptual Dysregulation	Type 1 vs. Type 2	00.083	16,899.5	0.022	0.165
	DD <2 vs. DD >2	–00.333	77,280.0	0.000	0.438
Unusual Beliefs and Experiences	Type 1 vs. Type 2	00.000	15,691	0.360	0.094
	DD <2 vs. DD >2	–00.143	113,820.5	0.000	0.239
Attention Seeking	Type 1 vs. Type 2	00.250	16,864.5	0.022	0.166
	DD <2 vs. DD >2	00.000	138,330.5	0.098	0.104
Callousness	Type 1 vs. Type 2	00.000	14,380	0.775	0.015
	DD <2 vs. DD >2	–00.167	119,000.0	0.000	0.212
Depressivity	Type 1 vs. Type 2	00.111	17,062	0.015	0.175
	DD <2 vs. DD >2	–00.556	60,900.0	0.000	0.528
Hostility	Type 1 vs. Type 2	00.333	17,402.5	0.005	0.194
	DD <2 vs. DD >2	–00.500	80,760.5	0.000	0.418
Perseveration	Type 1 vs. Type 2	00.250	17,418	0.005	0.195
	DD <2 vs. DD >2	–00.500	81,540.5	0.000	0.413
Restricted Affectivity	Type 1 vs. Type 2	00.250	15,833.5	0.347	0.102
	DD <2 vs. DD >2	–00.250	110,140.5	0.000	0.258
Rigid Perfectionism	Type 1 vs. Type 2	00.250	17,341	0.007	0.190
	DD <2 vs. DD >2	–00.375	113,530.5	0.000	0.238
Risk Taking	Type 1 vs. Type 2	00.125	16,578	0.053	0.146
	DD <2 vs. DD >2	00.125	162,730.5	0.552	0.032
Submissiveness	Type 1 vs. Type 2	00.250	169,31	0.022	0.167
	DD <2 vs. DD >2	–00.500	97,530.5	0.000	0.327
Suspiciousness	Type 1 vs. Type 2	00.250	17,128.5	0.013	0.179
	DD <2 vs. DD >2	–00.500	84,340.5	0.000	0.401
